# Easily processable spin filters: exploring the chiral induced spin selectivity of bowl-shaped chiral subphthalocyanines†

**DOI:** 10.1039/d3sc01069d

**Published:** 2023-04-03

**Authors:** Jorge Labella, Deb Kumar Bhowmick, Anil Kumar, Ron Naaman, Tomás Torres

**Affiliations:** a Department of Organic Chemistry, Universidad Autónoma de Madrid Campus de Cantoblanco, C/Francisco Tomás y Valiente 7 28049 Madrid Spain tomas.torres@uam.es; b Department of Chemical and Biological Physics, Weizmann Institute of Science 7610001 Israel ron.naaman@weizmann.ac.il; c Institute for Advanced Research in Chemical Sciences (IAdChem), Universidad Autónoma de Madrid 28049 Madrid Spain; d IMDEA-Nanociencia Campus de Cantoblanco 28049 Madrid Spain

## Abstract

High spin polarization (SP) in studies of chiral induced spin selectivity (CISS) is only observed when chiral molecules are properly organized. This is generally achieved by using anchoring groups or complex supramolecular polymers. A new class of spin filters based on bowl-shaped aromatics is reported, which form high-quality thin-films by simply spin-coating and displaying high spin filtering properties. In particular, we fabricate devices containing enantiopure tribromo-subphthalocyanines (SubPcs), and measure the CISS effect by means of magnetic conductive probe atomic force microscopy (mc-AFM). Circular dichroism and AFM experiments reveal that the resulting thin-film presents a well-ordered chiral structure. Remarkably, the resulting devices show SPs as high as *ca.* 50%, which are comparable to those obtained by using the current complex methodologies. These results boost the potential of bowl-shaped aromatics as easily processable spin filters, opening new frontiers toward realistic and efficient spintronic devices based on the CISS effect.

## Abstract 2

Herein a new class of spin filters based on subphthalocyanines is reported. We measure the CISS effect by means of magnetic conductive probe atomic force microscopy (mc-AFM). Remarkably, the resulting devices show spin polarizations (SPs) as high as *ca.* 50%.

## Graphical abstract

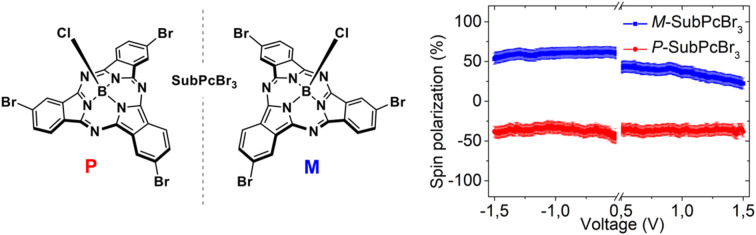

## Introduction

Spintronics, the combination of spin properties with electronics, is an attractive approach due to its potential in reducing noise and energy consumption in transferring and manipulating information.^1^ In recent years, it has been widely demonstrated that certain chiral organic molecules are able to produce spin polarized currents when electrons flow through them. In other words, chiral molecules can serve as spin filters. This fascinating property is known as chirality-induced spin selectivity (CISS) and, in a matter of few years, has become a hot topic because of its ability to produce large spin polarizations (SPs) even at room temperature.^2–4^ The CISS effect has also found important applications in many other fields, such as catalysis,^5^ enantio-separation,^6^ long range electron transfer,^7^ and bio-recognition.^8^

**Fig. 1:**
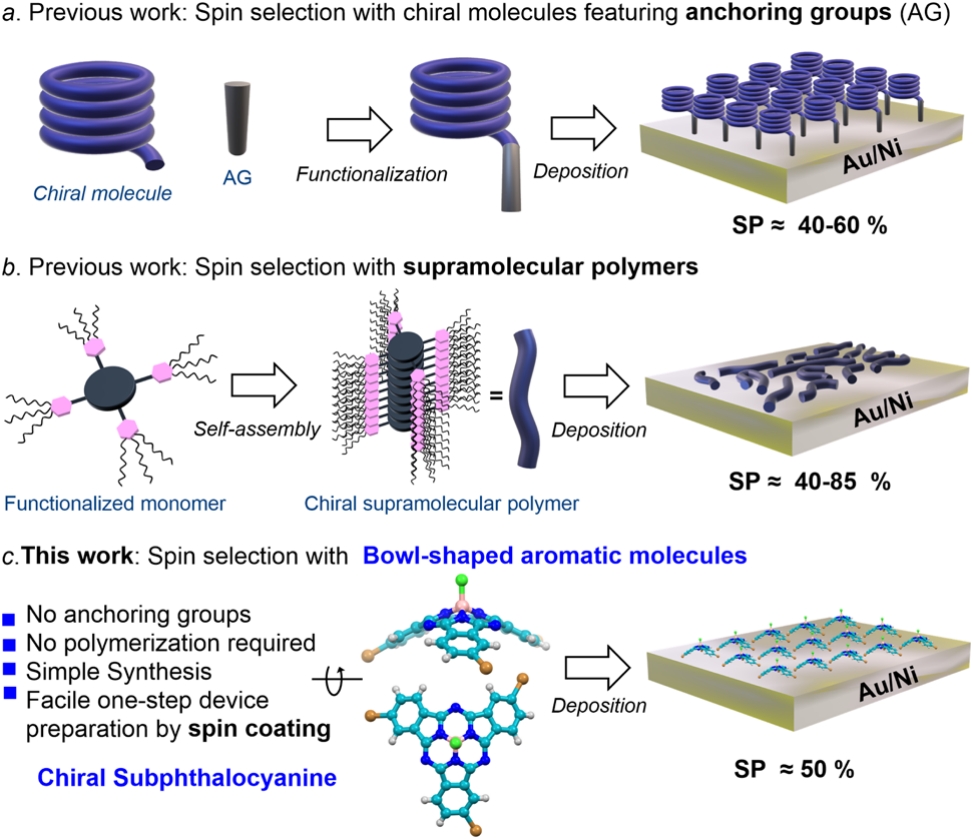
Existing strategies to prepare well-ordered chiral assemblies for mc-AFM measurements: (a) formation of SAMs. (b) Deposition of supramolecular polymers. (c) Methodology reported in this work: spin-coating deposition of bowl-shaped SubPcs (atom's colour code: carbon in turquoise, nitrogen in blue, hydrogen in white, boron in pink, chlorine in green and bromine in orange).

The CISS effect has been studied by means of numerous experimental techniques,^9–14^ and has been explored in multiple systems, including biomolecules (*e.g.*, DNA, peptides, and proteins),^15–17^ helicenes^18–20^ or chiral polymers.^21^ Among the most employed techniques, magnetic conductive probe atomic force microscopy (mc-AFM) actually holds a privileged position by virtue of its numerous advantages. As discussed below, mc-AFM measurements involve the deposition of chiral molecules on a gold-coated nickel surface (Au/Ni). The way in which the molecules are organized on the Au/Ni surface strongly affects the spin filtering efficiency. For obtaining reproducible results and in order to find correlation between molecular properties and spin filtering, the molecules must be organized in a well-defined way on the surface. The control of molecular orientation, however, is far from trivial given the entropy factor that leads to the creation of disorder in the adsorbed layer. In order to ensure organization, researchers have followed two main strategies (Fig. 1a and b): (a) the preparation of self-assembled monolayers (SAMs) employing chiral molecules equipped with anchoring groups (*e.g.*, –SH or –OH)^9,18,21–23^ and (b) the use of flat, π-conjugated molecules (*e.g.*, porphyrins and polycyclic hydrocarbons) able to self-assemble into chiral, columnar stacks.^24,25^ In the first case, the spin selectivity is measured along the main molecular axis, which is perpendicular to the substrate plane. By contrast, in method (b) the polymers lay on the Au/Ni surface and the CISS effect is measured perpendicular to the main molecular axis. These methods, although efficient and elegant, present major drawbacks. On one hand, method (a) requires the post- or pre-synthetic functionalization of the chiral molecule, which introduces complexity to the synthesis. Moreover, the number of molecules compatible with this technique is limited. On the other hand, method (b) requires a careful design and extensive characterization of the supramolecular polymer. In addition, the orientation of such polymers on the surface is problematic since the surface is not fully covered and measurements depend on the paths of the electrons. Taking these issues into account, the search for novel spin filters comprising a correct balance between easy processability and good performance is an essential step for bringing the CISS effect to practical devices.

Subphthalocyanines (SubPcs) are a well-known class of organic semiconductors displaying an unusual bowl-shaped aromatic skeleton.^26,27^ Due to their excellent physical, optical, and electronic properties, as well as their outstanding thin-film morphology in layered substrates, SubPcs have been skillfully applied in multiple technologies, including organic and perovskite solar cells (OSCs and PSCs),^28–32^ OLEDs,^33,34^ or photodetectors,^35^ just to name a few. Importantly, when properly functionalized, SubPcs can be chiral, and the corresponding enantiomers are configurationally stable and thus manipulable.^36–38^ In spite of this, the application of chiral SubPcs is still virgin territory. Just very recently, we have observed that enantiopure *C*_3_-symmetric SubPcs spontaneously form well-ordered chiral assemblies on Au(111) surfaces.^38^ This finding led us to envisage that thin-films based on enantiopure SubPcs could meet the organization criteria for efficient spin selection, without the need for anchoring groups or supramolecular polymerization. Herein we thus study the use of chiral SubPcs as a new class of easily processable spin filters. To this end, we fabricate devices where the SubPc is simply deposited by spin-coating and measure the CISS effect by means of mc-AFM. Circular dichroism and AFM experiments reveal that the resulting thin-film presents a well-ordered chiral structure. Remarkably, the resulting devices show SPs as high as *ca.* 50%, which are comparable to those obtained by using the current complex methodologies.

## Results and discussion

**Fig. 2:**
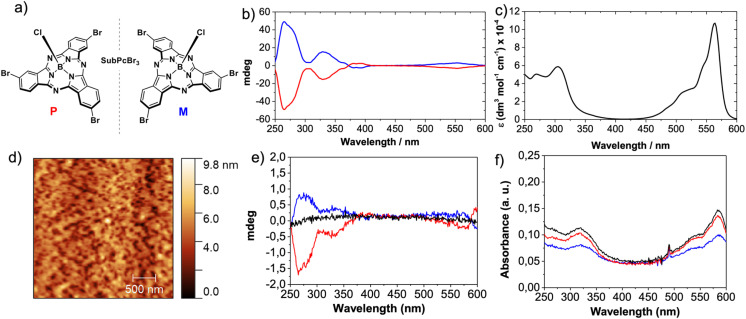
(a) Molecular structures of *P*- and *M*-SubPcBr_3_. (b) Circular dichroism spectra of enantiopure *M*-SubPcBr_3_ (blue spectrum) and *P*-SubPcBr_3_ (red spectrum) in toluene (concentration = 2 × 10^−5^ M). (c) UV-vis absorption spectra of enantiopure *M*-SubPcBr_3_ (blue spectrum) and *P*-SubPcBr_3_ (red spectrum) in toluene (concentration = 7 × 10^−6^ M). (d) AFM image of the substrate on which the *M*-SubPcBr_3_ molecules were deposited *via* spin-coating. (e) Solid-state CD spectra measured on quartz substrates of spin-coated enantiopure *M*-SubPcBr_3_ (blue spectrum), *P*-SubPcBr_3_ (red spectrum) and *rac*-SubPcBr_3_, and (f) the absorption spectrum of respective surfaces.

We selected SubPcBr_3_ enantiomers (*M*- and *P*-SubPcBr_3_; Fig. 2a) by virtue of their (i) excellent organization in the solid-state and, as previously mentioned, on Au surfaces and (ii) strong Cotton effects in circular dichroism (CD), which frequently anticipate an efficient CISS effect.^24^*M*- and *P*-SubPcBr_3_ were obtained by means of chiral HPLC according to our reported procedure, and the enantiopurity of the eluted compounds was confirmed by in-solution CD (Fig. 2b). With both enantiomers in hand, we embarked on the preparation of layered devices for mc-AFM measurements. It is well-known that high-quality thin-films based on SuPcs can be prepared by means of multiple techniques.^39^ Thus, given the good solubility of SubPcBr_3_ and the multiple advantages of solution-processing, we were focused on spin-coating deposition. After screening several conditions, we found that toluene solutions of SubPcBr_3_ led to the best results. The morphology of the resulting thin-films was studied by AFM. As shown in the top-view AFM images (Fig. 1d and the ESI†), rac-SubPcBr3, *M*- and *P*-SubPc-Br_3_ form smooth and uniform thin-films, with an excellent substrate covering and no evidence of any aggregation of the material.

Further insights into the thin-film organization were provided by solid-state UV-vis absorption spectroscopy (Fig. 2f). Compared to the UV-vis absorption in solution (Fig. 2c), the SubPc absorption bands were broadened and red-shifted in the thin-film (10 nm). Moreover, the Q-band exhibits higher quenching compared to the Soret band. These features point to the formation of columnar H-type-like aggregates in the solid state.^32,38^

Nevertheless, significant changes in the spectral shape were not observed, suggesting that the aggregation is not as marked as, for example, that of their phthalocyanine (*Pc*) homologues.^40^ Then, we performed solid-state CD in order to evaluate the chiral assembly of the spin-coated self-assembled layers. As shown in Fig. 2e, the thin-films based on *M*- and *P*-SubPc-Br_3_ showed mirror image CD spectra with strong Cotton effects that range from 200 to 350 nm. These features are in accordance with those observed in solution. As expected, the racemic compound (*rac*-SubPcBr_3_) showed no CD signal. Based on these results, we can conclude that *M*- and *P*-SubPc-Br_3_ form chiral self-assemblies on solid substrates which are exactly opposite to each other.

**Fig. 3:**
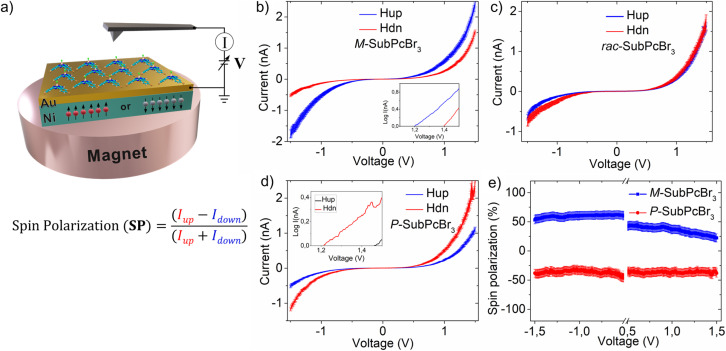
Spin-dependent transport properties measured with mc-AFM. (a) mc-AFM setup in which SubPcBr_3_ is deposited on the ferromagnetic substrate by spin-coating. (b), (c), and (d) present the averaged current *versus* voltage (*I*–*V*) curves recorded for *M*-, *rac*- and *P*-SubPcBr_3_, respectively. The blue line represents the current for the SubPc layer when the magnet north pole is pointing up and red line for the magnet north pole pointing down with respect to the substrate. (e) Spin polarization percentage (SP%) as a function of applied bias for *M*-SubPcBr_3_ (blue) and *P*-SubPcBr_3_ (red).

Next, we explored the CISS effect by mc-AFM. A scheme representing the mc-AFM setup used in this study is shown in Fig. 3a. The chiral SubPcs were deposited *via* spin-coating on a gold-coated nickel surface (Au/Ni), which was magnetized perpendicularly to the surface, with the north pole pointing either up or down. Depending on the direction of the external magnetic field, the spin of the ejected electrons from the nickel substrate to the deposited chiral molecular layer varies. The electric potential was applied so that the AFM tip was at ground, while the potential on Au/Ni was varied. In this way, the spin selective electron transport was readily measured and quantified (*i.e.*, SP) as the relative ratio of currents with the two different configurations of the magnet (up and down), that is, [SP= (*I*_up_ − *I*_down_)/(*I*_up_ + *I*_down_)] × 100.

The results obtained from the mc-AFM experiments are presented in Fig. 3b–e. As shown in the current–voltage curves (*I*–*V*; Fig. 3b–d), the current displayed by both *M*- and *P*-SubPcBr_3_ strongly depends on the direction of the magnetization of the substrate. Specifically, the *M*-SubPcBr_3_ enantiomer exhibits higher currents when the substrate is magnetized with the magnetic field up. In contrast, in the case of *P*-SubPcBr_3_ the current is lower with the same magnetization. Each curve is the average of over 50 individual measurements. The semi log plots presented in the insets show a nonlinear dependence of the current on the applied potential and a difference in the threshold for the currents of the electrons with the two-spin polarization, which is characteristic of chiral systems and indicates no spin flipping during electron transmissions.^41^ The racemic compound does not show any spin preferred current, as shown in Fig. 3c. Therefore, we can conclude that enantiopure SubPcBr_3_ displays spin-selective charge transport. Subsequently, we quantified these spin-filtering properties by calculating the SP percentage. The dependence of SPs on the applied voltage is shown in Fig. 3e. Remarkably, average SPs as high as +48 ± 5% and 45 ± 5% were obtained for *M*- and *P*-SubPcBr_3_, respectively. These values are very high considering that there are no anchoring groups or supramolecular polymerization, which highlights the excellent capability of SubPcs to self-organize in thin-films. It is important to appreciate that this value is comparable to those obtained from previously reported – and more complex – methods. For instance, method (a) (*i.e.*, self-assembled monolayers) leads to SPs in the range of 40–60%. Notably, in the case of helicenes the spin-coating deposition of derivatives non-functionalized with anchoring groups produces SPs below 5%. This value, however, rose up to 50% upon functionalization with thiols and preparation of SAMs. On the other hand, method (b) (*i.e.*, prior formation of supramolecular polymers) produces SPs between 40% and 85%, depending on the strategy to achieve the chiral assembly (*e.g.*, the use of chiral side chains, chiral solvents, *etc.*).

## Conclusions

In conclusion, we revealed the CISS effect of chiral SubPcs and its potential for spintronic devices. Although rationally controlling the organization of chiral molecules is still challenging, our result indicates that bowl-shaped π-conjugated molecules can form well-ordered, chiral thin-films with enough quality to serve as efficient spin filters, without the need of anchoring groups of complex supramolecular polymerization. We believe that this result will promote the study of the CISS effect of bowl-shaped aromatics and paves the way for realistic and efficient spintronic devices based on the CISS effect.

## Author contributions

R. N. and T. T. designed the research. J. L. performed the synthesis and studied the thin-film formation of chiral SubPcs. D. K. B. and A. K. performed the device preparation, characterization and mc-AFM experiments. All authors contributed to the writing and editing of the paper. Overall, J. L., D. K. B. and A. K. contributed equally to this publication.

## Conflicts of interest

The authors declare no competing financial interest.

## Supplementary Material

SC-014-D3SC01069D-s001
