# Supplementary material for: Easily processable spin filters: exploring the chiral induced spin selectivity of bowl-shaped chiral subphthalocyanines
Source: Chem Sci. 2023 Apr 3;14(16):4273–7. doi: 10.1039/d3sc01069d (PMC10132120; doi:10.1039/d3sc01069d)
Supplement: SC-014-D3SC01069D-s001 [file SC-014-D3SC01069D-s001.pdf]

Electronic Supplementary Material (ESI) for Chemical Science.
This journal is © The Royal Society of Chemistry 2023

Supplementary Information

Easily Processable Spin Filters: Exploring the Chiral Induced Spin Selectivity of Bowl-Shaped Chiral Subphthalocyanines.

Jorge Labella,[a‡] Deb Kumar Bhowmick,[b‡] Anil Kumar,[b‡] Ron Naaman,[b,*] Tomás Torres[a,c,d*].

[a] Departamento de Química Orgánica, Universidad Autónoma de Madrid, Madrid, 28049, Spain.

[b]Department of Chemical abd Biological Physics, Weizmann Institute of Science, 7610001, Israel.

[c] Institute for Advanced Research in Chemical Sciences (IAdChem), Universidad Autónoma de Madrid, Madrid, 28049, Spain.

[d] IMDEA-Nanociencia, Campus de Cantoblanco, Madrid, 28049, Spain.

[‡] These authors contributed equally to this work.

Abbreviations:

CD = circular dichroism; HPLC = high performance liquid chromatography; HR-MS = high resolution mass spectrometry; MALDI-TOF = matrix-assisted laser desorption/ionization-time of flight; MS = mass spectrometry; NMR = nuclear magnetic resonance; ppm = part per million; SubPc = subphthalocyanine; TLC = thin layer chromatography; UV-vis = ultraviolet-visible; THF = tetrahydrofuran; Mp= melting point; DCTB = *trans*-2-[3-(4-*tert*-Butylphenyl)-2-methyl-2-propenylidene]malononitrile.

1. Synthesis and characterization of Chiral SubPc

Chemicals were purchased from commercial suppliers and used without further purification unless otherwise stated. Thin layer chromatography (TLC) was carried out employing aluminium sheets coated with silica gel type 60 F254 (0.2 mm thick, E. Merck). Purification and separation of the C_1 and C_3 regioisomers of the synthesized products was performed by column chromatography using silica gel (230–400 mesh, 0.040–0.063 mm, Merck). Matrix Assisted Laser Desorption/ Ionization-Time of Flight (MALDI-TOF) spectra were obtained using a Bruker Ultraflex III spectrometer equipped with a Nd-YAG laser operating at 337 nm. High Resolution Mass Spectrometry (HR-MS) spectra were recorded employing ESI Positive Q-TOF using a Bruker Maxis II. The different matrixes employed are indicated for each spectrum. Mass spectrometry data are expressed in m/z units. ^{1}H-NMR spectra were recorded on a Bruker AC-300 (300 MHz) at room temperature. In the ^{1}H-NMR spectra, the chemical shifts (δ) are measured in ppm relative to the correspondent deuterated solvent. UV-Vis spectra were recorded employing a JASCO-V660 spectrophotometer. CD spectra were recorded with a JASCO V-815 equipment. Resolution of racemic $SubPcBr_3$ was carried out by chiral HPLC using an Agilent 1200 equipment with a semi-preparative Daicel Chiralpak IC column (10 mm ø x 20 mmL).

<u>Synthesis and characterization of $SubPcBr_3$.[1]</u>

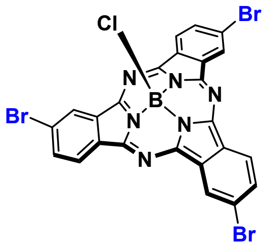

In a 50 mL two-necked round-bottomed flask, equipped with a condenser, magnetic stirrer and rubber seal, a 1.0 M solution of BCl_3 in p-xylene (7.25 mL) was added to 4-bromophthalonitrile (1.5 g, 7.25 mmol) under argon atmosphere. The reaction mixture was stirred at 140 ºC for 20 min. The purple solution was allowed to cool to room temperature and flushed with argon. The dark purple reaction slurry was dissolved in toluene/THF 10:1 and passed through a short silica plug. The solvent was removed by vacuum distillation and the resulting dark solid was purified by column chromatography on silica gel using toluene/heptane 3:1 as eluent. During the process, C_3 and C_1 regioisomers were separated. Upon recrystallization from methanol, 120 mg (0.18 mmol) and 410 mg (0.61 mmol) of C_3 and C_1 regioisomers, respectively, were obtained as purple-gold solids. Yield: 33%. **Mp** > 250 °C; **^{1}H-NMR** (300 MHz, $CDCl_3$): δ (ppm) = 9.01

[1] J. Labella, G. Lavarda, L. Hernández-López, F. Aguilar-Galindo, S. Díaz-Tendero, J. Lobo-Checa, T. Torres, *J. Am. Chem. Soc.* **2022**, *144*, 16579–16587.

(d, $^{4}J_{H-H}$ = 1.7 Hz, 3H), 8.72 (d, $^{3}J_{H-H}$ = 9.7 Hz, 3H), 8.05 (dd, $^{3}J_{H-H}$ = 9.7 Hz, $^{4}J_{H-H}$ = 1.7 Hz, 3H); **^{13}C-NMR** (75.5 MHz, $CDCl_3$): δ (ppm) = 150.3, 149.7, 134.2, 132.9, 130.1, 126.1, 125.6, 124.3; **^{11}B-NMR** (160.5 MHz, $CDCl_3$): δ (ppm) = -13.8 (s); MS (MALDI-TOF, DCTB): m/z = 667.9 [M]+. HRLSI-MS: m/z Calcd for [$C_{24}H_9BBr_3ClN_6$]: 667.8178; Found: 667.8195. **UV/vis** ($CHCl_3$): λ_{max} (nm) (log ε) = 562 (5.0), 305 (4.8).

With the racemic of $SubPcBr_3$ in hand, the corresponding enantiomers (***P***- and ***M*-SubPcBr$_3$**) were obtained by HPLC following the reported method previously reported by our group.[1]

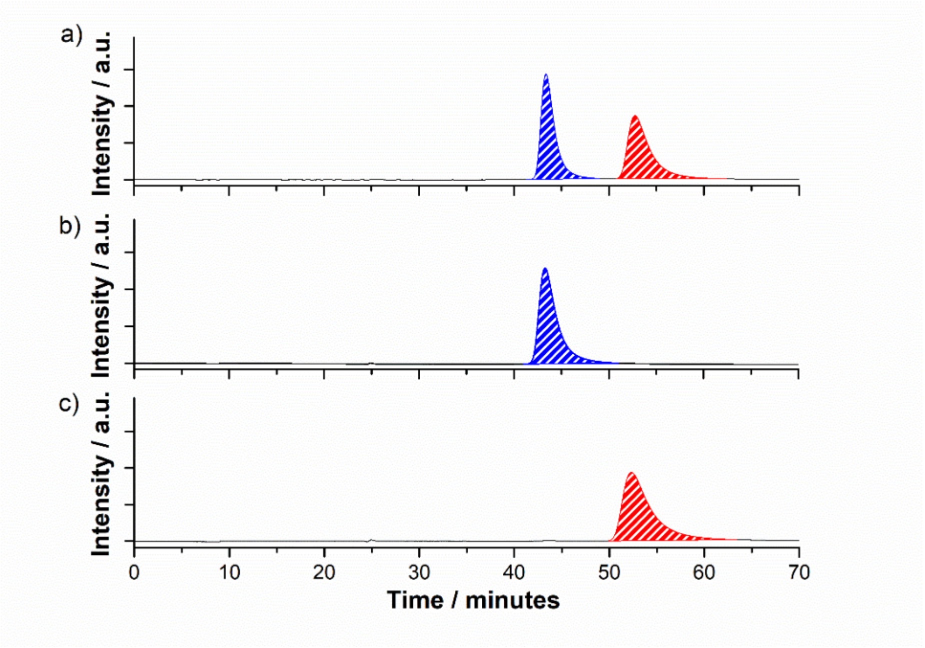

Figure S1. a) HPLC chromatogram of a) racemic $SubPcBr_3$ with peaks corresponding to *M* enantiomer (blue trace) and *P* enantiomer (red trace). The percentage area underneath the first and second peak is 50.0% and 50.0%, respectively. b) HPLC chromatograms of enantiopure $SubPcBr_3$. c) HPLC chromatograms of enantiopure $SubPcBr_3$. Eluting solvents = toluene/n-hexane 50:50; flow rate = 1.0 mL min−1; temperature = 10 ºC, detection wavelength = 570 nm

2. Thin-film preparation and magnetic-conductive atomic force microscopy (mc-AFM) experiments.

Thin-film preparation

The substrates for the mc-AFM studies were prepared using the e-beam evaporation deposition technique on Si (100) wafer. An 8 nm Ti layer followed by a 60 nm layer of Ni layer and a 5 nm layer of Au layer was deposited on a Si wafer. Thin gold layers were deposited to protect Ni layer from oxidizing. The deposited multilayer surfaces were cleaned by immersing them first in acetone and then in ethanol for 10 minutes. The role of the Ni/Au surfaces is to allow spin polarization of electrons injected from the surface into the chiral molecules induced by a magnetic field. The SubPc layer were deposited on the Ni/Au surfaces by spin coating the substrate with a corresponding compound solution of 0.5 mg /ml in toluene at 4000 rpm for 40 sec. Further, substrates were heated at a temperature of 125°C for 10 min and cooling substrates to room temperature at a cooling rate of 10°C/min.

mc-AFM experiments:

Magnetic field-dependent current-voltage (I-V) characteristics of the prepared samples were determined using a multimodal scanning magnetic probe microscopy (SPM) system equipped with a Beetle Ambient AFM and an electromagnet with an R9 electronic controller (RHK Technology). Voltage spectroscopy for the I-V measurements was performed by applying voltage ramps with a non-magnetic Pt tip (DPE-XSC11, μmasch with spring constant 3-5 Nm-1) in contact mode. At least 50 I-V curves were scanned in an applied magnetic field of 0.50 T for both magnetic field orientations (field UP and DOWN), and representative plots for (P) and (M) enantiomers on Ni/Au and ITO surfaces are depicted in Figure S1 and S2, respectively.

Spin dependent transport properties for (M) and (P) enantiomers on Ni/Au surfaces:

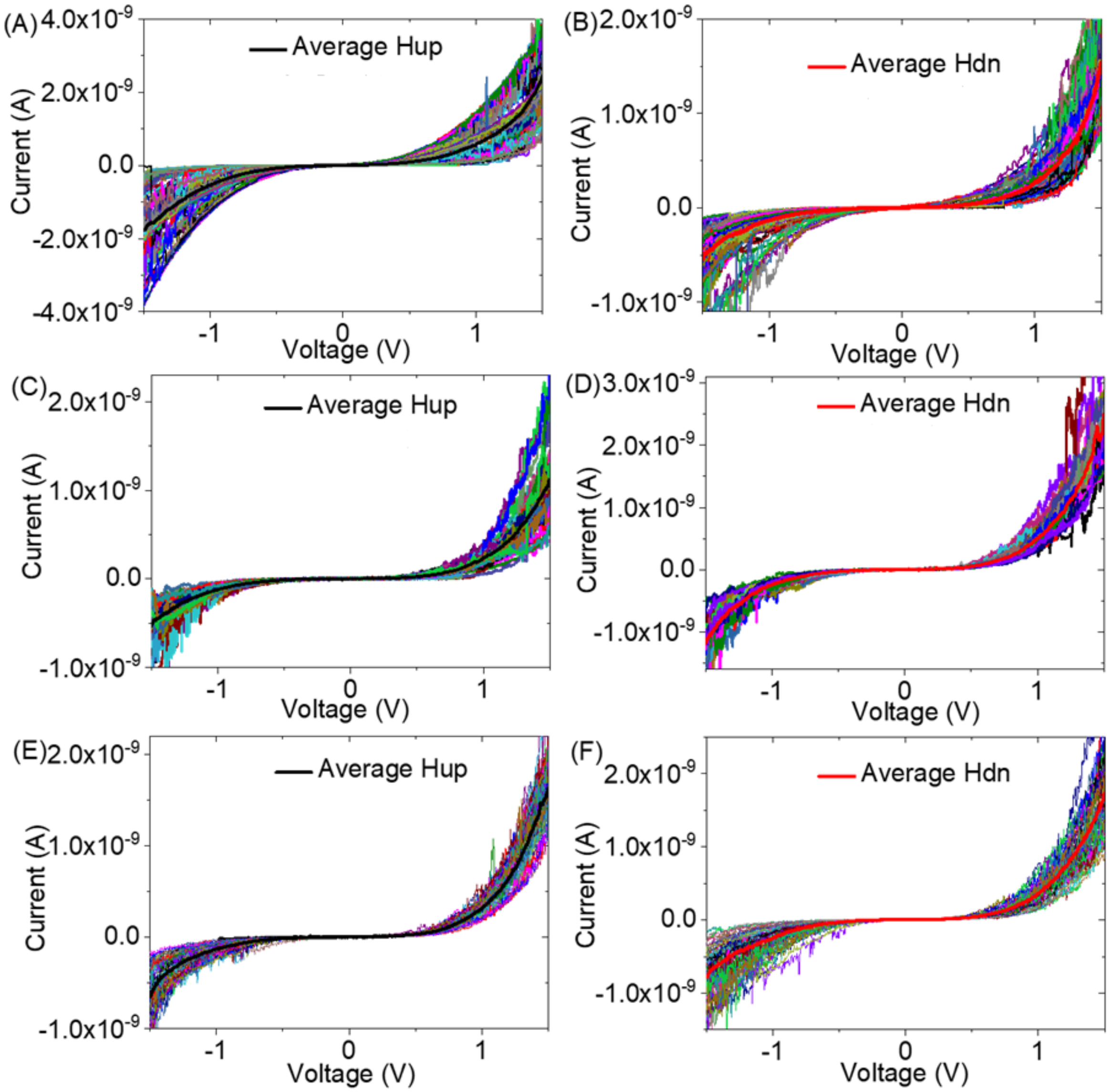

Figure S2. Current versus voltage (I-V) plots recorded for spin-coated (a-b) ***M*-**, and (c-d) ***P*-SubPcBr$_3$**, (e-f) ***rac*-SubPcBr$_3$** on Ni-Au substrates. In all cases, the Ni substrates were magnetized with the north pole pointing in the up (the black line) and down (the red line) orientations. The width of the lines (in multiple colours) represents the standard deviation of the measurements.

Preparation of Substrate for Solid State Circular Dichroism (CD) Measurements

The substrates for CD measurements were prepared spin coating of respective compound solutions in toluene as described previously for mc-AFM measurements. Before spin coating, the substrates were cleaned by immersing them first in aqua regia, followed by washing them in boiling water. Further, substrates were cleaned in boiling acetone and then in ethanol for 10 minutes each.
